# Evidence-based health policy in Germany: lack of communication and coordination between academia and health authorities?

**DOI:** 10.1186/s13643-023-02204-6

**Published:** 2023-03-13

**Authors:** Charlotte Mareike Kugler, Matthias Perleth, Tim Mathes, Kaethe Goossen, Dawid Pieper

**Affiliations:** 1 Faculty of Health Sciences Brandenburg, Brandenburg Medical School (Theodor Fontane), Institute for Health Services and Health System Research, Seebad 82/83, 15562 Rüdersdorf bei Berlin, Germany; 2 Center for Health Services Research, Brandenburg Medical School (Theodor Fontane), Seebad 82/83, 15562 Rüdersdorf bei Berlin, Germany; 3 Federal Joint Committee (G-BA), Gutenbergstraße 13, 10587 Berlin, Germany; 4 Department of Medical Statistics, University Medical Center Göttingen, Humboldtallee 32, 37073 Göttingen, Germany; 5 Witten/Herdecke University, Institute for Research in Operative Medicine, Ostmerheimer Straße 200, Haus 38, 51109 Köln, Germany

**Keywords:** Replication, Communication, Collaborative research, Knowledge translation

## Abstract

Health-care decision making should consider the best available evidence, often in the form of systematic reviews (SRs). The number of existing SRs and their overlap make their identification and use difficult. Decision makers often rely on de novo SRs instead of using existing SRs. We describe two cases of duplicate reviews (minimum volume threshold of total knee arthroplasties and lung cancer screening) and one case of duplicate primary data analysis (transcatheter aortic valve implantation). All cases have in common that unintended duplication of research occurred between health authorities and academia, demonstrating a lack of communication and coordination between them.

It is important to note that academia and health authorities have different incentives. Academics are often measured by the number of peer-reviewed publications and grants awarded. In contrast, health authorities must comply with laws and are commissioned to deliver a specific report within a defined period of time. Most replication is currently unintended. A solution may be the collaboration of stakeholders commonly referred to as integrated knowledge translation (IKT). The IKT approach means that research is conducted in collaboration with the end users of the research. It requires active collaborations between researchers and decision-makers or knowledge users (clinicians, managers, policy makers) throughout the research process. Wherever cooperation is possible in spite of requirements for independence or confidentiality, legal regulations should facilitate and support collaborative approaches between academia and health authorities.

## Background

### Epidemiology of systematic reviews

Health-care decision making should consider the best available evidence. In the spirit of evidence-based medicine, it is recommended to obtain such evidence by relying on high-quality systematic reviews (SRs).

The number of published SRs is growing exponentially: In 2010, Bastian et al. [1] published their remarkable study entitled “Seventy-five trials and eleven SRs a day: how will we ever keep up?”. Other authors have subsequently attempted to estimate the number of SRs [2–4], and while they sometimes differed in their prevalence estimates, they always agreed on the trend for exponential increase. The most recent such study found that nearly 80 SRs were published daily in 2019 [5].

Not surprisingly, this comes at the cost of overlapping SRs, i.e., multiple SRs dealing with the same or similar question [6, 7]. Replication is desirable to some extent [8], and multiple SRs on the same question reaching the same conclusion will increase confidence [9]. However, overlap leaves SR users with two major challenges. First, SRs can reach different conclusions [10–12]. Second, the reporting quality as well as the methodological quality of SRs still leaves a lot of room for improvement (e.g., [13]). Clearly spoken, there is a considerable amount of research waste in the conduct of SRs [14]. From a decision-maker’s perspective, this can be a very convoluted scenario. Should I use an already published SR? Is it reliable, methodologically sound, and does it address my specific research question? How do I choose one among multiple SRs? The risk of bias tool in systematic reviews can be helpful in answering some, but not all of these questions [15]. However, if one is not able or willing to rely on other authors’ SRs, there is no other choice than conducting a de novo SR, and by this potentially contributing to research waste. Notably, a recent review found that researchers make no or poor use of SRs when justifying and/or designing new studies, or when placing new study results in the context of existing similar research [16].

This article aims at encouraging a discussion on unintended duplication of systematic reviews or analyses by different health authorities and academia resulting from poor communication and coordination between the two worlds. For illustration, three examples are presented that raise the question of useful replication or research waste.

### Health authority organisations in Germany

This section presents German health authority organisations, which is relevant for understanding the following three cases. The *Federal Joint Committee* is the highest decision-making body in the German health care system. It regularly commissions the *Institute for Quality and Efficiency in Health Care (IQWiG)* with systematically reviewing the evidence on drugs, diagnostic, or therapeutic procedures as a basis for their decision-making purposes. IQWiG is scientifically independent. There is a legal foundation in the German Social Code Book V (the law for the German public health insurance system) of this sharing of responsibilities. Decisions made by the Federal Joint Committee are sometimes subject of court proceedings. IQWiG’s rigorous approach has resulted in the recognition of the trustworthiness of their evidence reports by German jurisdictions. IQWiG preferably synthesises evidence from RCTs and other clinical trials according to their own methodology instead of relying on already existing evidence syntheses. This inevitably increases the number of SRs and thus risk of duplicate SRs. In addition, there is no pre-registration of IQWiG reviews in databases of SRs, such as PROSPERO (but review protocols are published on the website). As part of its reviews, IQWiG also searches for pre-existing SRs and refers to them in the results or even uses them instead of primary studies; however, IQWiG is required by law to perform independent assessments (and reviews) with participation of clinical or methodological experts and as a matter of fact, usually relies on primary trial results. *The Institute for Quality Assurance and Transparency in Health Care (IQTIG)* is responsible for statutory quality assurance in the health care system. The Federal Joint Committee commissions IQTIG for the development and implementation of quality assurance procedures. *The Federal Office for Radiation Protection* is responsible for the safety and protection against radiation damage to the environment and population health. Therefore, its mandate includes assessing health-related risks from radiation (e.g., X-ray diagnostics) to advise the Federal Ministry for the Environment and other health authorities.

Below, the authors present three cases of unintended, publicly funded, duplication.

### Total knee arthroplasty and the minimum volume-threshold (first case)

One example for unintended duplication of evidence synthesis by academia and health authorities is related to “minimum volume standards” and the hospital volume-outcome relationship for total knee arthroplasties. There was no high-quality SR on this topic and thus no evidence for a minimum volume standard. Yet, German hospitals need to perform at least 50 total knee arthroplasties annually to get reimbursement for this procedure [17]. Therefore, the Institute for Research in Operative Medicine at Witten/Herdecke University received a grant notice in 2018 by the Ministry for Research and Education to conduct a SR. Although the review was registered on PROSPERO [18] and a protocol was published in 2020 [19], the Federal Joint Committee commissioned IQWiG to investigate the same question within a rapid report in 2021. Rapid reports by IQWiG aim to provide up-to-date information on current relevant topics and are comparable to rapid reviews. In the same year, the SR conducted at Witten/Herdecke University was published online [20], while IQWiG’s report was completed and published in 2022 without referencing the former [21]. With only minor differences in eligibility criteria, both reviews drew the same conclusions. On the one hand, the example can be seen as a case of unintended duplication of efforts because both reviews relied on the same set of primary studies, albeit the total number was lower in the IQWiG report due to stricter inclusion criteria. On the other hand, there have been many political debates on minimum volume standards and two reviews by independent institutions may strengthen the conclusions. In addition, different intentions and legal pathways may explain the case. Nevertheless, better communication and coordination between both institutions could increase synergies.

### Lung cancer screening (second case)

Various randomized controlled trials (RCTs) on lung cancer screening in heavy smokers have been completed within the last years. In many of these studies, computed tomography (CT) was found to reduce lung cancer mortality. SRs and clinical guidelines therefore recommended low-dose-CT screening [22]. The topic was also picked up in Germany, but the regulatory framework is complex: In the first step, any new screening intervention that uses X-rays needs to be formally approved by the Federal Office for Radiation Protection. This requires a risk-benefit assessment including modeling of long-term radiation safety issues [23]. In the second step, any new screening intervention has to be assessed in order to decide on statutory reimbursement and establishment of a screening programme by IQWiG. Both evaluation steps include a systematic assessment of risks and benefits. Accordingly, two SRs on the same topic were prepared within a few years by official agencies—first the Federal Office for Radiation Protection, then IQWiG. In the case of lung cancer screening, both assessments were based on the same set of primary studies and resulted in nearly identical effectiveness results [24, 25].

Unintended duplication of efforts could potentially have been avoided if both agencies (Federal Office for Radiation Protection and IQWiG) had better communication processes in place, allowing them to join efforts. From a scientific perspective, it would require fewer resources either if both agencies collaborated directly, or if one agency built on the previous work by the other. However, legal reasons require that both assessments are done independently from each other. Issues of confidentiality and independence allow only minimal scientific exchange between both agencies. The current workflow is stipulated by national law, which follows the principle of independent assessments without specifically aiming at replication of results.

### Transcatheter aortic valve implantation (third case)

Another example for unnecessary duplicate work in the context of evidence syntheses is the analysis of the relationship between volume and outcome in transcatheter aortic valve implantation (TAVI). In contrast to the other examples, it does not refer to multiple SRs but to primary data analyses.

Specifically, in 2021, IQWiG published an evidence report on the association between hospital volume and quality for TAVI [26]. The available evidence was not conclusive regarding all outcomes of interest. In addition, because no “German” study was included, it was doubted that the results were applicable to the German healthcare context. For this reason, the German Society of Cardiologists decided to verify the findings and gain additional insight for the German context. For this purpose, it was obvious that it would be best to use the German external quality assurance data, which cover all hospitals in Germany. The data can be analyzed by any researcher on reasonable request and are kept by IQTIG. A committee of the Federal Joint Committee must approve all proposals. The protocol by the German Society of Cardiologists for the analysis was submitted to IQTIG in May 2022 and accepted in July [27]. In the same month, the Federal Joint Committee itself commissioned the IQTIG to perform an analysis on the same question [28]. The analysis appears to be an unnecessary duplication of research because the same data are used and consequently, differences can arise only because of differences in the analysis methods. Both analyses are not yet published. The Federal Joint Committee can commission its own analyses instead of relying on already existing evidence syntheses to obtain the analyses that perfectly fits its decision problems. Therefore, this duplication can be considered to be unintended in the sense that it was not a planned replication.

In the sense of evidence-based research, the example may be viewed positively, as it was performed in response to a research gap identified in an evidence synthesis. However, it illustrates that research to fill such knowledge gaps should be coordinated.

## Discussion

We described two cases of duplicate reviews (minimum volume threshold of total knee arthroplasties and lung cancer screening) and one case of duplicate primary data analysis (TAVI) as additional analyses for an evidence synthesis. All cases have in common that two institutions (unintentionally) performed very similar analyses, where one analysis could have easily replaced the other, at least partly, to inform decision-making. Duplication of reviews occurs in other countries as well. Screening mammography, for example, was covered by two funded reviews (with slightly different questions) [29, 30], although an updated Cochrane review was available at the time [31]. This demonstrates a lack of communication and coordination between academia and health authorities and also between different health authorities. It is important to note that academia and health authorities have different incentives. Academics are often measured by the number of peer-reviewed publications (preferably in high impact journals) and attraction of grants [32]. In contrast, health authorities must comply with laws and are commissioned to conduct a certain report usually according to a strictly defined methodology within a defined period of time, so that academia and health authorities also have different timelines [33]. While scientists often work in projects for several years, health authorities need more rapid information to rely on in their decision marking. This means that although they might be working on the same topic, academics and health authorities belong to two distinct worlds.

There are certain reasons to consider several high-quality publications as useful replication and reasons that justify the conduct of more than one SR [34]. If several publications confirm each other’s findings, this can also be seen positively, as it provides a firmer basis for decision-making. Replication definitely has its place in the context of SRs. However, replication can be either unintended or intended. Most replication is currently unintended. There is also a need to discuss where replication ends and research waste begins.

To prevent unintended duplication, which can contribute to research waste in some cases, institutions and academia first need to be aware of each other’s ongoing and planned work and to use prior SRs when planning new studies [16]. Evidence-based research requires a systematic search before starting an analysis. A second step is deciding whether the question (e.g., the PICO) is the same or sufficiently similar and whether available evidence syntheses can be used. Quality, up-to-dateness or applicability of findings to one’s own local context are frequent barriers. In addition, registration is necessary to identify ongoing studies. But at the moment, health institutions do not regularly register their SRs or analyses. At the same time, legal or regulatory restrictions prevent them from relying on (published) evidence or from collaborating. Therefore, regulations would need to be adjusted so that health authorities can make use of existing evidence syntheses or primary analyses. However, the law requires that key decisions in healthcare are based on analyses that fulfil specific criteria with regard to independence, transparency, patient involvement, and public commenting opportunities. SRs from academia seldom fulfil all of these criteria. Accordingly, health authorities currently use academic SRs mainly as a quarry from which to extract building blocks for their own SRs [35].

A solution may be collaboration of stakeholders commonly referred to as integrated knowledge translation (IKT). The IKT approach means that research is conducted in collaboration with those who will use the results of the research (end-users). Therefore, IKT requires active collaboration between researchers and decision-makers or knowledge users (clinicians, managers, policy-makers, etc.) throughout the research process [36]. Benefits from IKT include research questions being more relevant for practice or policy, findings being more easily adapted and implemented into practice, and an increased understanding of the different roles [37]. There are contextual factors influencing IKT practice at the organizational and individual level. These factors influence the impact of IKT on research, social, and health services outcomes [33]. Collaboration may not only be relevant between researchers and health authorities within the same country, but also between authorities of different countries, e.g., for the assessment of surgical procedures, new medicines and medical devices. According to the European Union’s Regulation on Health Technology Assessment [38], Member States’ HTA bodies will begin in 2025 to conduct Joint Clinical Assessments of new medicines and certain high-risk medical devices. This regulation as well as IKT strategies of other countries shows that collaboration between different organization is possible. We argue to strengthen such collaborations in Germany.

## Conclusion

Research waste is known to be an issue in particular with SRs. Intended replication makes sense, while unintended duplication should be avoided. Collaborative approaches are needed to improve the situation. Awareness of ongoing (or even better: planned) projects is a prerequisite to this. Where possible, legal regulations need to allow for and facilitate collaborative approaches between academia and health authorities as well as between different health authorities.

## Data Availability

n.a.

## Abbreviations

CT: Computed tomography
IKT: Integrated knowledge translation
IQTIG: Institute for Quality Assurance and Transparency in Health Care
IQWiG: Institute for Quality and Efficiency in Health Care
SR: Systematic review
TAVI: Transcatheter aortic valve implantation
